# 2-[5-(2-Fluoro­phen­yl)-3-isobutyl-1*H*-pyrazol-1-yl]benzoic acid

**DOI:** 10.1107/S1600536812050702

**Published:** 2013-01-04

**Authors:** S. Sreenivasa, K. E. Manojkumar, P. A. Suchetan, N. R. Mohan, Vijith Kumar, B. S. Palakshamurthy

**Affiliations:** a Department of Studies and Research in Chemistry, Tumkur University, Tumkur, Karnataka 572 103, India; b Department of Studies and Research in Chemistry, U.C.S., Tumkur University, Tumkur, Karnataka 572 103, India; c Soild State and Structural Chemistry Unit, Indian Institute of Science, Bangalore 560 012 India; d Department of Studies and Research in Physics, U.C.S., Tumkur University, Tumkur, Karnataka 572 103, India

## Abstract

In the title compound, C_20_H_19_FN_2_O_2_, the dihedral angle between the aromatic rings is 62.1 (1)°, and those between the pyrazole ring and the fluoro­benzene and benzoic acid rings are 52.1 (1) and 53.1 (1)°, respectively. In the crystal, mol­ecules are linked into [010] *C*(7) chains by O—H⋯N hydrogen bonds.

## Related literature

For background to pyrazole derivatives and their uses, see: Ramaiah *et al.* (1999 ▶).
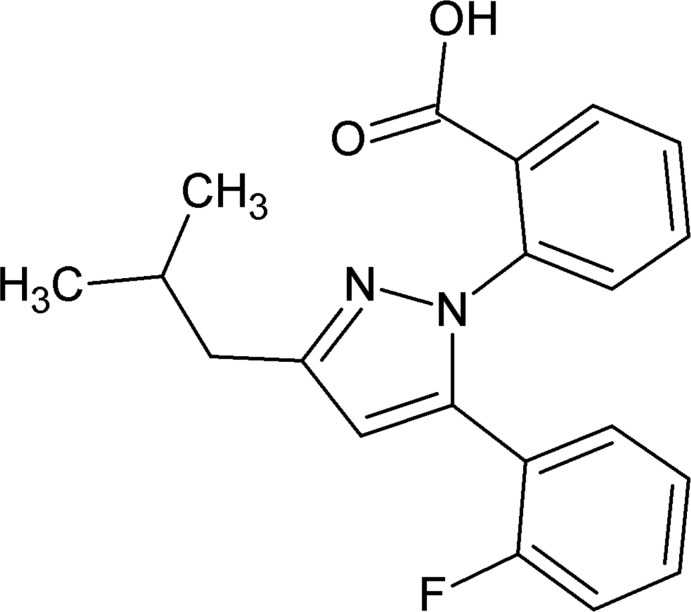


## Experimental

### 

#### Crystal data

C_20_H_19_FN_2_O_2_
*M* *_r_* = 338.37
Monoclinic, 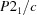

*a* = 9.7732 (14) Å
*b* = 12.2671 (16) Å
*c* = 15.257 (2) Å
β = 106.836 (5)°
*V* = 1750.7 (4) Å^3^
*Z* = 4
Mo *K*α radiation
μ = 0.09 mm^−1^
*T* = 293 K
0.28 × 0.26 × 0.22 mm

#### Data collection

Bruker SMART X2S CCD diffractometer
Absorption correction: multi-scan (*SADABS*; Bruker, 2004 ▶) *T*_min_ = 0.975, *T*_max_ = 0.980
16424 measured reflections
3094 independent reflections
2517 reflections with *I* > 2σ(*I*)
*R*_int_ = 0.042

#### Refinement

*R*[*F* ^2^ > 2σ(*F* ^2^)] = 0.041
*wR*(*F* ^2^) = 0.114
*S* = 0.98
3094 reflections
228 parameters
H-atom parameters constrained
Δρ_max_ = 0.23 e Å^−3^
Δρ_min_ = −0.21 e Å^−3^

### 

Data collection: *APEX2* (Bruker, 2004 ▶); cell refinement: *SAINT-Plus* (Bruker, 2004 ▶); data reduction: *SAINT-Plus*; program(s) used to solve structure: *SHELXS97* (Sheldrick, 2008 ▶); program(s) used to refine structure: *SHELXL97* (Sheldrick, 2008 ▶); molecular graphics: *ORTEP-3* (Farrugia, 2012 ▶); software used to prepare material for publication: *SHELXL97*.

## Supplementary Material

Crystal structure: contains datablock(s) I, global. DOI: 10.1107/S1600536812050702/hb7011sup1.cif

Structure factors: contains datablock(s) I. DOI: 10.1107/S1600536812050702/hb7011Isup2.hkl

Supplementary material file. DOI: 10.1107/S1600536812050702/hb7011Isup3.cml

Additional supplementary materials:  crystallographic information; 3D view; checkCIF report

## Figures and Tables

**Table 1:** Hydrogen-bond geometry (Å, °)

*D*—H⋯*A*	*D*—H	H⋯*A*	*D*⋯*A*	*D*—H⋯*A*
O1—H1⋯N2^i^	0.82	1.93	2.7118 (16)	159

Symmetry code: (i) 
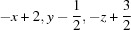
.

**Fig. 1.**
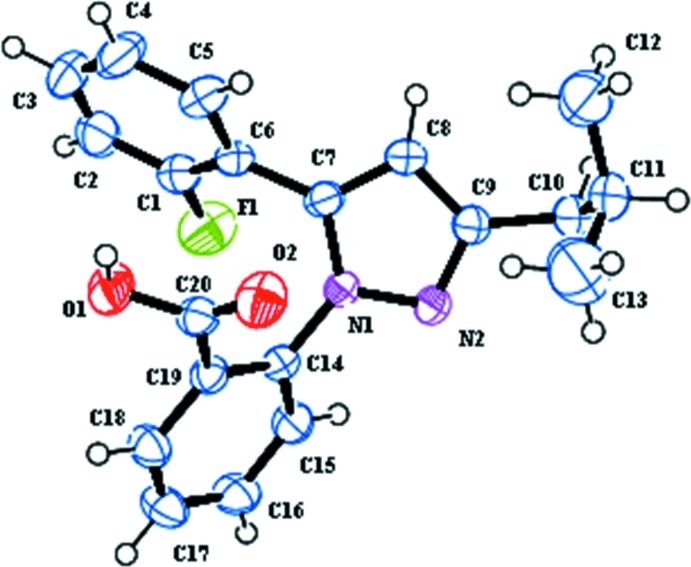
Molecular structure of the title compound, showing displacement ellipsoids drawn at the 50% probability level.

**Fig. 2.**
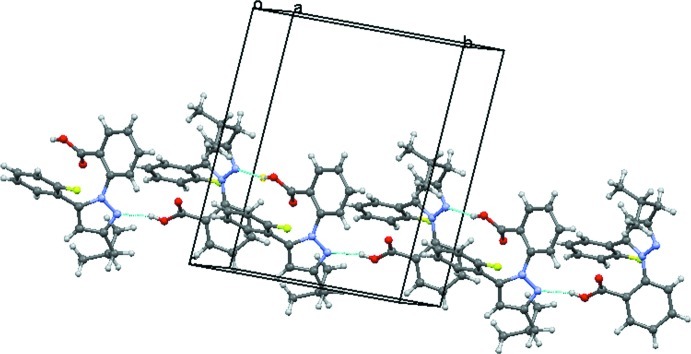
Molecular packing of the title compound.

## supplementary crystallographic information

### Comment

The pyrazole nucleus is an important structure in numerous natural and synthetic compounds and in medicinal chemistry (e.g. Ramaiah *et al.*, 1999). As part of our studies in this area, the title compound was synthesized and its crystal structure determined.

In the title compound, C_20_H_19_FN_2_O_2_, the dihedral angle between the aromatic rings is 62.1 (1)°, and those between the pyrazole ring and the aromatic ring containing fluorine atom and the pyrazole ring and the aromatic ring containing carboxylic group are 52.1 (1)° and 53.1 (1)°, respectively. In the crystal structure, the molecules are linked into C(7) chains through O—H···N H-bonds.

### Experimental

1-(2-Fluorophenyl)-5-methyl-hexane-1,3-dione (0.01 mmol) and 2-hydrazinobenzoic acid (0.01 mmol) were taken in 15 ml ethanol and the mixture was heated for 12 h. The reaction was monitored by TLC. The solvent was removed by vacuum. The crude mass obtained was purified by column chromatography.

Colourless prisms were obtained by slow evaporation of the solution of the compound in a mixture of dichloromethane and methanol (9:1).

### Refinement

The H atoms were positioned with idealized geometry using a riding model with C—H = 0.93- 0.97 Å. All C—H atoms were refined with isotropic displacement parameters (set to 1.2–1.5 times of the *U*_eq_ of the parent atom) and O—H atoms were refined freely

### Crystal data

**Table:**

C_20_H_19_FN_2_O_2_	Prism
*M**_r_* = 338.37	*D*_x_ = 1.284 Mg m^−^^3^
Monoclinic, *P*2_1_/*c*	Melting point: 514 K
Hall symbol: -P 2ybc	Mo *K*α radiation, λ = 0.71073 Å
*a* = 9.7732 (14) Å	Cell parameters from 228 reflections
*b* = 12.2671 (16) Å	θ = 2.2–25°
*c* = 15.257 (2) Å	µ = 0.09 mm^−^^1^
β = 106.836 (5)°	*T* = 293 K
*V* = 1750.7 (4) Å^3^	Prism, colourless
*Z* = 4	0.28 × 0.26 × 0.22 mm
*F*(000) = 712	

### Data collection

**Table:**

Bruker SMART X2S CCD diffractometer	3094 independent reflections
Radiation source: fine-focus sealed tube	2517 reflections with *I* > 2σ(*I*)
Graphite monochromator	*R*_int_ = 0.042
Detector resolution: 1.20 pixels mm^-1^	θ_max_ = 25.0°, θ_min_ = 2.2°
ω scans	*h* = −11→11
Absorption correction: multi-scan (*SADABS*; Bruker, 2004)	*k* = −14→13
*T*_min_ = 0.975, *T*_max_ = 0.980	*l* = −13→18
16424 measured reflections	

### Refinement

**Table:**

Refinement on *F*^2^	Primary atom site location: structure-invariant direct methods
Least-squares matrix: full	Secondary atom site location: difference Fourier map
*R*[*F*^2^ > 2σ(*F*^2^)] = 0.041	Hydrogen site location: inferred from neighbouring sites
*wR*(*F*^2^) = 0.114	H-atom parameters constrained
*S* = 0.98	*w* = 1/[σ^2^(*F*_o_^2^) + (0.0699*P*)^2^ + 0.3164*P*] where *P* = (*F*_o_^2^ + 2*F*_c_^2^)/3
3094 reflections	(Δ/σ)_max_ < 0.001
228 parameters	Δρ_max_ = 0.23 e Å^−^^3^
0 restraints	Δρ_min_ = −0.21 e Å^−^^3^
0 constraints	

### Special details

**Table:**

Geometry. All e.s.d.'s (except the e.s.d. in the dihedral angle between two l.s. planes) are estimated using the full covariance matrix. The cell e.s.d.'s are taken into account individually in the estimation of e.s.d.'s in distances, angles and torsion angles; correlations between e.s.d.'s in cell parameters are only used when they are defined by crystal symmetry. An approximate (isotropic) treatment of cell e.s.d.'s is used for estimating e.s.d.'s involving l.s. planes.
Refinement. Refinement of *F*^2^ against ALL reflections. The weighted *R*-factor *wR* and goodness of fit *S* are based on *F*^2^, conventional *R*-factors *R* are based on *F*, with *F* set to zero for negative *F*^2^. The threshold expression of *F*^2^ > σ(*F*^2^) is used only for calculating *R*-factors(gt) *etc*. and is not relevant to the choice of reflections for refinement. *R*-factors based on *F*^2^ are statistically about twice as large as those based on *F*, and *R*- factors based on ALL data will be even larger.

### Fractional atomic coordinates and isotropic or equivalent isotropic displacement parameters (Å^2^)

**Table:**

	*x*	*y*	*z*	*U*_iso_*/*U*_eq_	
C1	0.67611 (17)	0.18408 (13)	0.80869 (11)	0.0433 (4)	
C2	0.57943 (19)	0.10016 (18)	0.78124 (12)	0.0593 (5)	
H2	0.4818	0.1141	0.7611	0.071*	
C3	0.6306 (2)	−0.00440 (16)	0.78423 (13)	0.0650 (6)	
H3	0.5671	−0.0621	0.7658	0.078*	
C4	0.7749 (2)	−0.02427 (14)	0.81427 (13)	0.0610 (5)	
H4	0.8088	−0.0953	0.8160	0.073*	
C5	0.86956 (18)	0.06077 (13)	0.84190 (12)	0.0477 (4)	
H5	0.9671	0.0463	0.8628	0.057*	
C6	0.82182 (15)	0.16797 (12)	0.83906 (9)	0.0357 (3)	
C7	0.92409 (15)	0.25727 (11)	0.87236 (10)	0.0340 (3)	
C8	1.02695 (16)	0.26669 (12)	0.95519 (10)	0.0392 (4)	
H8	1.0485	0.2159	1.0024	0.047*	
C9	1.09279 (16)	0.36759 (12)	0.95454 (10)	0.0378 (4)	
C10	1.21498 (18)	0.41685 (14)	1.02714 (11)	0.0511 (4)	
H10A	1.1961	0.4095	1.0859	0.061*	
H10B	1.2195	0.4941	1.0147	0.061*	
C11	1.36062 (19)	0.36548 (15)	1.03400 (12)	0.0583 (5)	
H11	1.4327	0.4099	1.0773	0.070*	
C12	1.3732 (2)	0.25065 (17)	1.07315 (17)	0.0761 (6)	
H12A	1.3115	0.2026	1.0295	0.114*	
H12B	1.3458	0.2508	1.1287	0.114*	
H12C	1.4703	0.2261	1.0859	0.114*	
C13	1.3945 (3)	0.3702 (3)	0.94334 (17)	0.0942 (8)	
H13A	1.3313	0.3224	0.9003	0.141*	
H13B	1.4916	0.3478	0.9519	0.141*	
H13C	1.3821	0.4435	0.9203	0.141*	
C14	0.85515 (14)	0.37883 (11)	0.73233 (9)	0.0314 (3)	
C15	0.78672 (16)	0.47868 (12)	0.71577 (10)	0.0393 (4)	
H15	0.7900	0.5255	0.7643	0.047*	
C16	0.71348 (18)	0.50905 (13)	0.62735 (11)	0.0461 (4)	
H16	0.6707	0.5774	0.6164	0.055*	
C17	0.70354 (17)	0.43865 (14)	0.55532 (11)	0.0484 (4)	
H17	0.6529	0.4588	0.4960	0.058*	
C18	0.76933 (17)	0.33819 (13)	0.57190 (10)	0.0426 (4)	
H18	0.7602	0.2898	0.5236	0.051*	
C19	0.84933 (15)	0.30803 (11)	0.65991 (10)	0.0332 (3)	
C20	0.93555 (17)	0.20534 (11)	0.67171 (10)	0.0372 (4)	
N1	0.93084 (12)	0.35052 (9)	0.82486 (8)	0.0327 (3)	
N2	1.03412 (13)	0.41900 (9)	0.87541 (8)	0.0376 (3)	
O1	0.85946 (13)	0.12237 (8)	0.62925 (8)	0.0525 (3)	
H1	0.9097	0.0676	0.6370	0.079*	
O2	1.06073 (13)	0.20202 (9)	0.71308 (9)	0.0558 (3)	
F1	0.62576 (11)	0.28686 (9)	0.80786 (9)	0.0691 (3)	

### Atomic displacement parameters (Å^2^)

**Table:**

	*U*^11^	*U*^22^	*U*^33^	*U*^12^	*U*^13^	*U*^23^
C1	0.0448 (9)	0.0438 (9)	0.0405 (8)	−0.0036 (7)	0.0111 (7)	0.0071 (7)
C2	0.0467 (9)	0.0770 (14)	0.0486 (10)	−0.0214 (9)	0.0049 (8)	0.0101 (9)
C3	0.0784 (14)	0.0570 (12)	0.0561 (11)	−0.0366 (11)	0.0141 (10)	−0.0011 (9)
C4	0.0837 (14)	0.0359 (10)	0.0670 (12)	−0.0172 (9)	0.0278 (10)	−0.0013 (8)
C5	0.0543 (10)	0.0357 (9)	0.0553 (10)	−0.0059 (7)	0.0194 (8)	0.0044 (7)
C6	0.0424 (8)	0.0345 (8)	0.0312 (7)	−0.0066 (6)	0.0123 (6)	0.0035 (6)
C7	0.0395 (7)	0.0287 (7)	0.0352 (8)	−0.0021 (6)	0.0131 (6)	0.0026 (6)
C8	0.0459 (8)	0.0357 (8)	0.0341 (8)	−0.0034 (7)	0.0084 (6)	0.0070 (6)
C9	0.0454 (8)	0.0327 (8)	0.0326 (7)	−0.0025 (6)	0.0071 (6)	−0.0008 (6)
C10	0.0680 (11)	0.0402 (9)	0.0363 (8)	−0.0133 (8)	0.0013 (8)	−0.0011 (7)
C11	0.0505 (10)	0.0629 (12)	0.0505 (10)	−0.0212 (9)	−0.0026 (8)	0.0073 (9)
C12	0.0611 (12)	0.0631 (13)	0.0900 (16)	−0.0011 (10)	−0.0006 (11)	0.0129 (11)
C13	0.0682 (14)	0.141 (2)	0.0767 (15)	−0.0206 (15)	0.0256 (12)	0.0082 (15)
C14	0.0346 (7)	0.0262 (7)	0.0332 (7)	−0.0011 (6)	0.0095 (6)	0.0012 (6)
C15	0.0470 (8)	0.0290 (8)	0.0410 (8)	0.0044 (6)	0.0114 (7)	−0.0041 (6)
C16	0.0530 (9)	0.0337 (8)	0.0495 (9)	0.0118 (7)	0.0116 (7)	0.0075 (7)
C17	0.0515 (9)	0.0511 (10)	0.0376 (8)	0.0099 (8)	0.0048 (7)	0.0091 (7)
C18	0.0515 (9)	0.0414 (9)	0.0331 (8)	0.0039 (7)	0.0092 (7)	−0.0032 (7)
C19	0.0376 (7)	0.0275 (7)	0.0352 (7)	−0.0005 (6)	0.0117 (6)	−0.0016 (6)
C20	0.0500 (9)	0.0292 (8)	0.0336 (7)	0.0022 (7)	0.0140 (7)	−0.0009 (6)
N1	0.0405 (6)	0.0240 (6)	0.0314 (6)	−0.0026 (5)	0.0070 (5)	−0.0009 (5)
N2	0.0484 (7)	0.0258 (6)	0.0349 (7)	−0.0045 (5)	0.0062 (5)	−0.0029 (5)
O1	0.0647 (7)	0.0282 (6)	0.0586 (7)	0.0048 (5)	0.0083 (6)	−0.0092 (5)
O2	0.0485 (7)	0.0389 (7)	0.0751 (9)	0.0092 (5)	0.0103 (6)	−0.0032 (6)
F1	0.0516 (6)	0.0598 (7)	0.0958 (9)	0.0099 (5)	0.0212 (6)	0.0115 (6)

### Geometric parameters (Å, º)

**Table:**

C1—F1	1.3522 (19)	C11—H11	0.9800
C1—C6	1.378 (2)	C12—H12A	0.9600
C1—C2	1.378 (2)	C12—H12B	0.9600
C2—C3	1.373 (3)	C12—H12C	0.9600
C2—H2	0.9300	C13—H13A	0.9600
C3—C4	1.373 (3)	C13—H13B	0.9600
C3—H3	0.9300	C13—H13C	0.9600
C4—C5	1.377 (2)	C14—C15	1.3832 (19)
C4—H4	0.9300	C14—C19	1.394 (2)
C5—C6	1.392 (2)	C14—N1	1.4338 (17)
C5—H5	0.9300	C15—C16	1.383 (2)
C6—C7	1.471 (2)	C15—H15	0.9300
C7—N1	1.3659 (18)	C16—C17	1.379 (2)
C7—C8	1.373 (2)	C16—H16	0.9300
C8—C9	1.396 (2)	C17—C18	1.379 (2)
C8—H8	0.9300	C17—H17	0.9300
C9—N2	1.3352 (18)	C18—C19	1.394 (2)
C9—C10	1.501 (2)	C18—H18	0.9300
C10—C11	1.532 (3)	C19—C20	1.497 (2)
C10—H10A	0.9700	C20—O2	1.2039 (19)
C10—H10B	0.9700	C20—O1	1.3151 (18)
C11—C13	1.514 (3)	N1—N2	1.3669 (16)
C11—C12	1.521 (3)	O1—H1	0.8200
			
F1—C1—C6	118.34 (14)	C11—C12—H12A	109.5
F1—C1—C2	118.56 (15)	C11—C12—H12B	109.5
C6—C1—C2	123.09 (16)	H12A—C12—H12B	109.5
C3—C2—C1	118.49 (17)	C11—C12—H12C	109.5
C3—C2—H2	120.8	H12A—C12—H12C	109.5
C1—C2—H2	120.8	H12B—C12—H12C	109.5
C2—C3—C4	120.41 (16)	C11—C13—H13A	109.5
C2—C3—H3	119.8	C11—C13—H13B	109.5
C4—C3—H3	119.8	H13A—C13—H13B	109.5
C3—C4—C5	120.13 (18)	C11—C13—H13C	109.5
C3—C4—H4	119.9	H13A—C13—H13C	109.5
C5—C4—H4	119.9	H13B—C13—H13C	109.5
C4—C5—C6	121.12 (17)	C15—C14—C19	120.03 (13)
C4—C5—H5	119.4	C15—C14—N1	118.60 (12)
C6—C5—H5	119.4	C19—C14—N1	121.37 (12)
C1—C6—C5	116.77 (14)	C16—C15—C14	120.21 (14)
C1—C6—C7	122.82 (14)	C16—C15—H15	119.9
C5—C6—C7	120.32 (14)	C14—C15—H15	119.9
N1—C7—C8	106.43 (12)	C17—C16—C15	120.39 (14)
N1—C7—C6	124.95 (12)	C17—C16—H16	119.8
C8—C7—C6	128.60 (13)	C15—C16—H16	119.8
C7—C8—C9	106.51 (13)	C16—C17—C18	119.48 (14)
C7—C8—H8	126.7	C16—C17—H17	120.3
C9—C8—H8	126.7	C18—C17—H17	120.3
N2—C9—C8	110.30 (12)	C17—C18—C19	121.03 (14)
N2—C9—C10	121.24 (13)	C17—C18—H18	119.5
C8—C9—C10	128.43 (13)	C19—C18—H18	119.5
C9—C10—C11	114.19 (15)	C14—C19—C18	118.75 (13)
C9—C10—H10A	108.7	C14—C19—C20	122.46 (13)
C11—C10—H10A	108.7	C18—C19—C20	118.58 (13)
C9—C10—H10B	108.7	O2—C20—O1	125.16 (13)
C11—C10—H10B	108.7	O2—C20—C19	122.86 (13)
H10A—C10—H10B	107.6	O1—C20—C19	111.94 (13)
C13—C11—C12	112.3 (2)	C7—N1—N2	110.89 (11)
C13—C11—C10	111.49 (16)	C7—N1—C14	129.41 (11)
C12—C11—C10	112.06 (16)	N2—N1—C14	119.52 (11)
C13—C11—H11	106.8	C9—N2—N1	105.87 (11)
C12—C11—H11	106.8	C20—O1—H1	109.5
C10—C11—H11	106.8		
			
F1—C1—C2—C3	−178.45 (15)	C14—C15—C16—C17	2.4 (2)
C6—C1—C2—C3	0.0 (3)	C15—C16—C17—C18	−1.1 (3)
C1—C2—C3—C4	0.2 (3)	C16—C17—C18—C19	−2.0 (3)
C2—C3—C4—C5	0.2 (3)	C15—C14—C19—C18	−2.4 (2)
C3—C4—C5—C6	−0.8 (3)	N1—C14—C19—C18	177.45 (13)
F1—C1—C6—C5	177.89 (14)	C15—C14—C19—C20	172.29 (13)
C2—C1—C6—C5	−0.5 (2)	N1—C14—C19—C20	−7.9 (2)
F1—C1—C6—C7	1.4 (2)	C17—C18—C19—C14	3.7 (2)
C2—C1—C6—C7	−177.03 (15)	C17—C18—C19—C20	−171.20 (15)
C4—C5—C6—C1	0.9 (2)	C14—C19—C20—O2	−46.5 (2)
C4—C5—C6—C7	177.51 (15)	C18—C19—C20—O2	128.16 (17)
C1—C6—C7—N1	−54.0 (2)	C14—C19—C20—O1	135.65 (15)
C5—C6—C7—N1	129.63 (16)	C18—C19—C20—O1	−49.67 (19)
C1—C6—C7—C8	124.18 (18)	C8—C7—N1—N2	−0.46 (16)
C5—C6—C7—C8	−52.2 (2)	C6—C7—N1—N2	178.04 (13)
N1—C7—C8—C9	0.22 (17)	C8—C7—N1—C14	174.51 (14)
C6—C7—C8—C9	−178.22 (14)	C6—C7—N1—C14	−7.0 (2)
C7—C8—C9—N2	0.10 (18)	C15—C14—N1—C7	130.38 (16)
C7—C8—C9—C10	−178.06 (16)	C19—C14—N1—C7	−49.4 (2)
N2—C9—C10—C11	−104.15 (18)	C15—C14—N1—N2	−55.02 (18)
C8—C9—C10—C11	73.8 (2)	C19—C14—N1—N2	125.15 (14)
C9—C10—C11—C13	56.3 (2)	C8—C9—N2—N1	−0.37 (17)
C9—C10—C11—C12	−70.6 (2)	C10—C9—N2—N1	177.94 (14)
C19—C14—C15—C16	−0.6 (2)	C7—N1—N2—C9	0.52 (16)
N1—C14—C15—C16	179.57 (14)	C14—N1—N2—C9	−175.02 (12)

### Hydrogen-bond geometry (Å, º)

**Table:**

*D*—H···*A*	*D*—H	H···*A*	*D*···*A*	*D*—H···*A*
O1—H1···N2^i^	0.82	1.93	2.7118 (16)	159

Symmetry code: (i) −*x*+2, *y*−1/2, −*z*+3/2.
